# Reduced costs of reproduction in females mediate a shift from a male-biased to a female-biased lifespan in humans

**DOI:** 10.1038/srep24672

**Published:** 2016-04-18

**Authors:** Elisabeth Bolund, Virpi Lummaa, Ken R. Smith, Heidi A. Hanson, Alexei A. Maklakov

**Affiliations:** 1 Department of Ecology and Genetics, Evolutionary Biology Centre, Uppsala University, Uppsala SE-752 36, Sweden; 2 Department of Biology, University of Turku, FIN-20014 Turku, Finland; 3 Department of Animal & Plant Sciences, University of Sheffield, Sheffield S10 2TN, UK; 4 Department of Family and Consumer Studies and Population Sciences, Huntsman Cancer Institute, University of Utah, Salt Lake City, UT 84112, USA; 5 Department of Family and Preventive Medicine and Population Sciences, Huntsman Cancer Institute, University of Utah, Salt Lake City, UT 84112, USA

## Abstract

The causes underlying sex differences in lifespan are strongly debated. While females commonly outlive males in humans, this is generally less pronounced in societies before the demographic transition to low mortality and fertility rates. Life-history theory suggests that reduced reproduction should benefit female lifespan when females pay higher costs of reproduction than males. Using unique longitudinal demographic records on 140,600 reproducing individuals from the Utah Population Database, we demonstrate a shift from male-biased to female-biased adult lifespans in individuals born before versus during the demographic transition. Only women paid a cost of reproduction in terms of shortened post-reproductive lifespan at high parities. Therefore, as fertility decreased over time, female lifespan increased, while male lifespan remained largely stable, supporting the theory that differential costs of reproduction in the two sexes result in the shifting patterns of sex differences in lifespan across human populations. Further, our results have important implications for demographic forecasts in human populations and advance our understanding of lifespan evolution.

Sex differences in lifespan are common across species, but which sex lives the longest varies among taxonomic groups^1^,^2^,^3^,^4^,^5^,^6^. The reasons for this are poorly understood, although several non-mutually exclusive hypotheses have been put forward^1^,^3^,^5^,^7^. Here we focus on the fact that the sexes often have different reproductive strategies, leading to different phenotypic trait optima^1^,^4^,^6^,^8^,^9^,^10^,^11^,^12^. The ‘disposable soma’ theory of ageing^13^,^14^,^15^,^16^ maintains that increased investment into reproduction accelerates senescence of the organism and shortens lifespan, because reproduction can cause direct damage to the soma and requires resources that could otherwise be used for somatic maintenance, such as cellular repair^13^,^14^. Strong evidence for a cost of reproduction has been found in a variety of taxa^3^,^15^,^17^, but such costs can differ between the sexes, because one sex often invests more into production and care of the offspring^4^. This can lead to different optimal trade-offs between reproduction and lifespan in the two sexes, resulting in sexual dimorphism in lifespan^6^. Support for this hypothesis comes mainly from studies on model organisms in laboratory settings (see^6^) and studies on populations outside the laboratory are needed to assess the generality of these findings across the tree of life^18^.

In humans, as in most mammals, women tend to outlive men in most countries and cultures^19^ and a female-biased lifespan is generally seen as the norm^19^,^20^. However, there is considerable variation in sexual dimorphism across time and space: a comparison of 227 human populations found substantial variation between populations, with some even showing male-biased lifespans^21^, and the life expectancy at birth was male-biased in two out of 208 countries in 2014^22^. A recent study of 13 industrialised populations concluded that a strongly female-biased lifespan is a recent phenomenon, prevalent only in people born after the late 1800s^23^. The reasons for such sex differences in human lifespan are of interest and importance across several disciplines, because they can impact demographic forecasts^24^, have implications for treatments in bio-gerontology and general medicine^25^, and further our understanding of lifespan evolution in general.

One little considered explanation for such variation in sex differences in human lifespan is the differing costs of reproduction affecting females and males at different times and in different populations. There is increasing evidence that many life-history traits are under different selection pressures in the two sexes in humans^26^,^27^,^28^. However, it remains debated whether costs of reproduction affect humans and how these differ between sexes, populations and across time. The few studies that have looked for costs of reproduction in men suggest that men bear low if any costs (refs 29, 30, 31, but see ref. 32). A large number of studies have looked for costs of reproduction in women, but the findings are highly mixed^30^,^33^,^34^,^35^,^36^,^37^. For example, the same historical dataset on women in Quebec, Canada, was used to support a positive relationship between total parity and post-reproductive survival^38^, a negative relationship^39^, or no relationship^40^. This lack of agreement may partly be due to differences in sampling procedures and methodologies, because a study that applied the same sample selection criteria and statistical analyses to three historical datasets (historical Quebec, recent Quebec and historical Utah) found a consistently negative relationship between female parity and post-reproductive survival in all three populations^30^.

Such differences in the costs of reproduction within and between populations may in part explain the different patterns of sexual dimorphism in lifespan. Indeed, a study on populations from 205 countries found that female birth rate explained 17% of the variance in relative sex differences in lifespan^41^. Importantly, over the last 200 years, many human populations have gone through the demographic transition to low birth and death rates^42^. This has led to changes in selection pressures on life history traits^43^,^44^,^45^,^46^ and likely radically altered life-history trade-offs in modern societies. A key example is the rapid decline in fertility rates that likely result in reduced physiological costs of reproduction, particularly in females, and thus an average resource allocation to reproduction that may be too low to constrain life-history allocations^15^,^36^. Nevertheless, despite the generally lowered fertility after the demographic transition and rising rates of nulliparity in modern nations, large variation remains, and the average number of children varies between slightly over one in contemporary Europe^21^ to around ten in the Hutterites, an Anabaptist group that practices communal living and shuns birth control^47^. Collectively, these previous findings suggest that, given the premise that reproduction is more costly in females than males, a recently reduced cost of reproduction has had a more pronounced effect on female life-history trade-offs, leading to a more female-biased longevity in modern populations. However, populations differ in many respects other than fertility levels that could potentially influence sexual dimorphism in lifespan. For example, patterns of alcohol consumption and infection pressures could explain part of the differences in sexual dimorphism in lifespan across latitudes and cultures^21^. Such differences between populations may result in spurious correlations if they are not identified and statistically controlled for. One way around this problem would be to quantify longitudinally any changes in sex differences in lifespan in a single population that experiences changes in the cost of reproduction. However, to our knowledge no study has tested whether changes in sexual dimorphism over time within a single population is related to costs of reproduction.

Here, we use a unique resource to study the patterns of survival and reproduction in the two sexes in a single population before, during and after the demographic transition. This allows us to document changes in the sexual dimorphism in lifespan over time in a population that is going through dramatic demographic changes and relate this to the costs of reproduction in the two sexes. The Utah Population Database (UPDB) is one of the world’s largest existing multigenerational research demographic datasets. The full UPDB now contains data on over 8 million individuals from the late 18th century to the present. The multigenerational pedigrees representing Utah’s founders and their descendants were constructed based on data provided by the Genealogical Society of Utah (GSU). Pedigrees spanning the past 80 years have been expanded extensively based on vital records and, together with the GSU data, form the basis of the deep genealogical structure of the UPDB. We use a subset of 140,600 reproducing individuals that had full information on their reproductive history as well as a number of demographic variables that can influence life-history patterns, such as birth place, birth order, being in a polygamous union and the identity of the birth mother. In historic Utah, fertility rates were very high, with average reproductive rates reaching nine children (Fig. 1) and this decreased dramatically over the demographic transition, which started around 1870–1880^48^. Specifically, we first quantify how sexual dimorphism in lifespan changed over time as the population went through the demographic transition. Second, we examine whether either sex payed a cost of reproduction in terms of a shortened post-reproductive lifespan. We predict that sexual dimorphism in adult lifespan should change over time if fertility patterns change.

## Results

### Sexual dimorphism in lifespan

**Figure 1:**
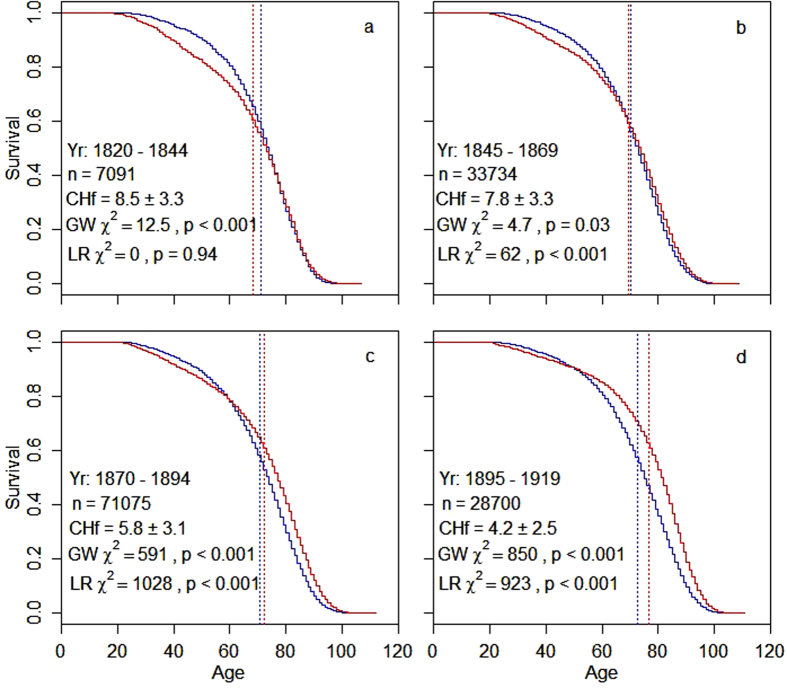
Age-specific survival after the onset of reproduction in four 25-year birth cohorts that cover the demographic transition in Utah. Survival curves represent females (red) and males (blue) that reproduced and had full known reproductive history. Shown in each figure are the birth years contained in that cohort (Yr), the sample size (n), the average ± SD number of children born to females (CHf), the ^χ2^ and p-values indicating differences in the survivor function between the two sexes from the Peto and Peto modification of the Gehan-Wilcoxon test (GW, this test weights differences in survivorship that occur early more heavily than differences at later survival times) and from a log-rank test (LR, this test weights differences at later survival times more heavily). Dotted vertical lines indicate the average lifespan of each sex in each cohort.

**Figure 2:**
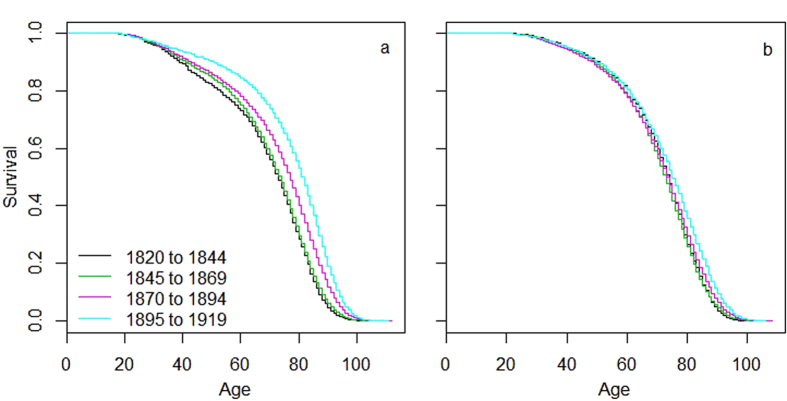
Change in adult survival over four 25-year birth cohorts. Survival curves represent females (**a**) and males (**b**) that reproduced and had full known reproductive history. Colours indicate the birth years contained in each cohort. For sample sizes, see Fig. 2.

We found clear evidence that sexual dimorphism in adult lifespan changed across the transition of the population from high to low mortality and fertility rates. Over the study period, which included individuals born between 1820 and 1919, average reproduction was halved from 8.5 to 4.2 children born per female and average adult lifespan increased by 12% in females and 2% in males (Fig. 1, Supplementary Table 1). Figure 1 shows the Kaplan-Meier survival curves and associated log-rank and Wilcoxon tests separately for the two sexes over four 25-year birth cohorts covering the demographic transition in Utah. The population changed from a significant male-biased adult survival in the earliest cohort to a progressively more female-biased adult survival in the later three cohorts. Parametric survival models that accounted for polygamy status, birth place, birth order and maternal identity showed a significant difference in the adult lifespan of the two sexes in all four cohorts (Table 1). The acceleration factor indicates the estimated mean survival time for females in relation to males in each cohort. Thus, males were predicted by the model to outlive females during adulthood by almost one year in the first cohort (birth years 1820–1844), the sexes had nearly identical predicted adult lifespans in the second cohort (1845–1869), and in the third (1870–1894) and fourth (1895–1919) cohorts, females were predicted to outlive males by about two and four years, respectively. Figure 2 illustrates the clear increase in female lifespan over time (2a) while male lifespan increased only marginally (2b, Supplementary Table 1).

In the early part of the 1800s a large proportion of the population suffered the hardships of migration into Utah. This may result in a robust surviving cohort and could have different effects on survival in the two sexes (a ‘healthy migrant’ effect^49^). The analyses detailed above includes all individuals with known birth and death information, regardless of whether they were born or died in Utah or elsewhere. To test for a healthy migrant effect in the early migrants, we compared individuals that migrated into Utah with individuals that spent their entire lives in Utah in birth cohort 2. This comparison showed that migration did not have a significant effect on survival in either sex during this time period (Supplementary Figure 1a, Parametric survival model: main effect estimate for the difference between migrants and non-migrants: −0.0005 ± 0.003, z = −0.16, p = 0.87, interaction estimate between sex and migratory status: −0.004 ± 0.004, −0.82, p = 0.41). Because the first birth cohort did not contain any individuals that were born in Utah, a similar comparison was not possible in this early time period. Instead, to validate the use of cohort 2 for comparing migrants to non-migrants, we compared migrants in cohort 2 with migrants in cohort 1 (Supplementary Figure 1b). This comparison showed that migration had similar effects on survival within each sex in these two time periods (parametric survival model: estimate for the difference between the two cohorts in males: −0.003 ± 0.004, z = −0.93, p = 0.35, and in females: 0.005 ± 0.004, z = 1.2, p = 0.22). A healthy migrant effect is thus unlikely to be a major contributor to the male-biased lifespan in the early cohort.

### Sex differences in the reproduction-longevity trade-off

**Figure 3:**
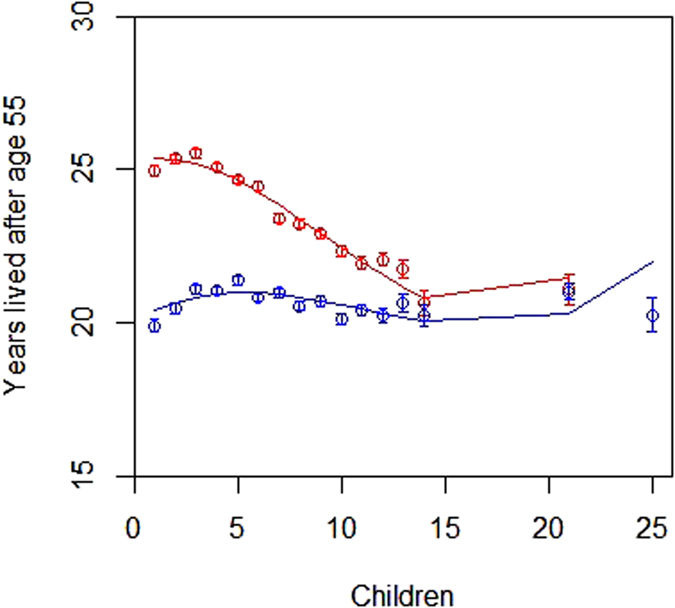
The number of years lived after the end of the potential reproductive period (age 55 years) related to the number of children born. Curves represent females (red) and males (blue) that reproduced and were born between 1820 and 1920. The raw data are grouped here for visual purposes only while analyses (see main text) are performed on ungrouped data and account for a number of fixed and random effects. Points with associated SE refer to grouped averages for all individuals of each sex at each parity. For ease of illustration, individuals are grouped at high parities into categories with 15–21 children and 22–65 children (only males, plotted at value 25 on the x-axis). Lines represent the best fit from third order polynomial regressions weighted by the sample size in each group (sample sizes range from 282 to 7264).

We investigated the potential causes for the changed sexual dimorphism in adult lifespan over the study period by measuring changes in the relationship between reproduction and lifespan after the end of the potential reproductive period at age 55 (post-reproductive lifespan) of each sex across the time period. Figure 3 shows the relationship between reproductive effort and post-reproductive lifespan in the two sexes, based on grouped raw data of 118,911 individuals. We estimated the differences between the sexes in the linear slopes of the relationship between number of children born and post-reproductive lifespan in models that accounted for polygamy status, birth place, birth order, birth cohort and maternal identity. This showed that there was a trade-off between reproduction and post-reproductive lifespan in females, but not in males (posterior mode for the interaction between sex and number of children born: −1.12, Bayesian credibility interval (CI) = −1.23 to −0.99, pMCMC = <0.001). This pattern was consistent across the four birth cohorts (The posterior modes for the interaction between sex and number of children born in four separate models, one for each birth cohort, ranged from −0.38 to −0.77). Separate models in each sex revealed opposing directions of the effect on post-reproductive lifespan in males and females of children ever born, with increasing parity being associated with decreased post-reproductive lifespan in women but increased post-reproductive lifespan in men (Sex-specific slopes: females: quadratic slope: −0.12, CI = −0.19 to −0.047, linear slope: −0.37, CI = −0.47 to −0.29, males: quadratic slope: −0.076, CI = −0.11 to −0.037, linear slope: 0.33, CI = 0.23 to 0.44). An alternative analysis approach, using parametric survival models, found consistent results. Models showed a slight increase in lifespan for females with two to four children compared to females with one child and thereafter a progressive decline in female lifespan at higher parities. The acceleration factor indicates that females in the highest parity group with 15 children or more were predicted to live about 6 years shorter than females with one child (Table 2). In contrast, male lifespan was little affected by the level of reproductive effort with a predicted difference in mean lifespan of less than one year in the different parity groups (Table 2).

## Discussion

In contrast to previous studies, which tend to treat time as a confounding variable (e.g.^38^), we here made use of the fact that the study population went through dramatic changes in fertility and lifespan over the study period, and investigated how this influenced the sexual dimorphism in adult lifespan. The average adult lifespan of the population increased over the demographic transition, mainly due to improvements in medicine and living conditions. Such improvements are likely to have similar positive effects on the lifespan of both sexes, yet we find a striking reversal from a male-biased to a female-biased adult lifespan in reproducing individuals over the span of only one century. Thus, the more pronounced increase in female adult lifespan compared to men during the second half of the study period must be due to other factors in addition to the increased living standard. Our focus on adult lifespan suggests that the shifting sexual dimorphism in lifespan is not due to potential shifts in gender biased infant mortality over time. Instead, we here argue that costs of reproduction is likely a contributing factor. These findings have implications for the predictions of future patterns of sexual dimorphism in human populations as global fertility patterns continue to change^24^. Further, biologically rooted differences between the sexes in lifespan are likely to be of importance for treatments in medicine, with special implications for bio-gerontology as we see global trends towards ageing populations^25^,^50^,^51^.

Reproductive rates were comparatively high in Utah before the demographic transition and showed a marked decrease from about 1880 onwards (Fig. 1). The higher female mortality in the early cohorts is mainly due to increased mortality during their reproductive years (Fig. 1). Women who died the same year in which they gave birth to their last child may often have died as a result of childbirth, and this proportion decreased over time (Supplementary Table 2). This may be explained by the higher average reproductive rate of the early time periods, because, given that each birth carries a small risk of maternal mortality, the cumulative risk of death that is directly caused by childbearing increases with increased parity^52^. In addition, medical advances in the later cohorts likely led to a much lower risk of death directly associated with childbirth. Crucially, we found that female lifespan beyond the reproductive years decreased with increasing parity (Fig. 3). Thus, it seems likely that high rates of reproduction not only carries a direct cost in terms of an increased risk of death as a result of childbearing^52^, but also carries physiological costs that result in a shortened lifespan due to trade-offs between reproduction and soma in females. This finding is consistent with the disposable soma theory of ageing^13^,^14^ and supports the hypothesis that sexual dimorphism in lifespan is the result of different optimal trade-offs between reproduction and survival in the two sexes^1^,^4^.

Our results are in line with the general pattern that sexual dimorphism in lifespan tends to increase with increasing average lifespan in the population^21^. Increased male mortality compared to females has been attributed to social factors, such as consumption of alcohol and other drugs^21^, and environmental factors, for example infections and other diseases in general affect men more strongly at all ages than women^19^. It has been suggested that stressful factors in general would influence men more negatively than women, thus increasing the excess of male mortality in stressful environments^53^. Contrary to this, we found that males outlived women before the demographic transition, when environmental conditions would have been more stressful. In addition, male lifespan increased only slightly over time. Nevertheless, there may have been other differences in living circumstances. For example, it is commonly observed that women living under more adverse conditions tend to have higher fertility but die at a younger age^54^. Therefore, while the different effects of childbearing on lifespan in the two sexes point to a role for biological effects, rather than purely social or economic factors^55^, a range of factors may contribute and warrant further study.

Our findings of sex-specific costs of reproduction build on the few previous studies that have compared the costs of reproduction in the two sexes in humans in general^29^,^39^ and the Utah population in particular^32^,^56^. Two previous studies on the UPDD found a cost of preproduction in females and no, or a low, cost of reproduction in males. Both studies focussed on couples who entered into marriage during a 35 to 40 year time period around the time of the demographic transition, and our study thus extends the time period under study to a 100-year period. Penn and Smith^56^ found that the costs of reproduction increased more with age in women than in men and that parity had an adverse effect on the survival of women, but not men, after age 50. Smith *et al*.^32^ found a similar effect, with a decreased lifespan after age 60 in women bearing six children or more compared to women with lower parities^32^. The same study found some cost of reproduction in men, although the husband’s longevity was less sensitive to reproductive history than their wife’s^32^.

The results of our study are subject to a few caveats. We limited our analyses to reproductive individuals because only reproducing individuals bear costs of reproduction. Individuals that did not reproduce, and hence did not suffer any costs of reproduction, showed markedly different patterns of sexual dimorphism in adult lifespan over time (Supplementary Fig. 2). Namely, adult lifespan was consistently female-biased, suggesting that the absence of a cost of reproduction leads to a female-biased longevity in all time periods. This finding further supports the notion that sex-specific costs of reproduction mediate shifting patterns of sex differences in mortality in this population. The data on non-reproductive individuals should, however, be interpreted with caution, because individuals may be classified as non-reproducers when there is a lack of data. Thus, this subset likely contains individuals that reproduced but did not have their reproduction recorded completely. Hence, we do not include formal analyses on these presumably non-reproductive individuals, but illustrate the patterns on the available raw data only in Supplementary Fig. 2. Further, by limiting our analyses of the relationship between completed fertility and lifespan to individuals that survived to the age of 55, we are potentially focussing on a biased subset of the population, consisting of the most robust individuals, because individuals that died as a result of childbearing or of other causes of death during middle adulthood were excluded. This would decrease our chances of observing a trade-off between fertility and lifespan if healthier, more robust individuals have both higher fertility and longer lifespans^15^,^57^. A previous study on the same population accounted for the effects of this type of mortality selection, and still found a cost of reproduction in terms of decreased post-reproductive survival in women as parity increased^30^. Furthermore, men are less susceptible to the physiological risks of childbearing and hence such a mortality selection effect is expected to be stronger in females. Despite this, we find a trade-off in women, but not in men. Similarly, the hardships of migration may result in a robust surviving cohort and could have different effects in the two sexes^49^. Contrary to this expectation, we found that the adult survival of individuals that migrated into Utah did not differ from individuals that spent their entire lives in Utah in individuals born between 1845 and 1869. The early immigrants to Utah (born 1820-1844) may have been subject to harsher conditions during migration, resulting in a healthy migrant effect. However, a comparison of early (born 1820–1844) and later (born 1845–1869) migrants into Utah showed no difference in the effects of migration on survival over time in women (Supplementary Fig. 2a). In men, there was a trend for higher survival in the early migrants, but this was not significant after accounting for fixed and random effects that can influence survival patterns (Supplementary Fig. 2b). These results suggest that, while the harsh conditions of the early migration may have to some extent selected for a more robust cohort of individuals, this did not have a strong effect on survival.

Patterns of resource acquisition vary dramatically both between and within human populations, and variation in acquisition can result in positive phenotypic correlations between life-history traits on an individual level if only low-acquisition individuals face a trade-off^15^,^58^. Thus, socioeconomic status (SES) is likely to influence costs of reproduction and lifespan, and the effects of SES may differ between the sexes^59^. However, previous studies on the UPDB that looked on the relationship between parity and parental longevity in both sexes found that patterns were not confounded by socioeconomic status^32^,^56^. Thus, while average socioeconomic status in the population likely changed over time, it seem unlikely that our results are biased by sex-specific responses to such changes. Because infant mortality in historical Utah was comparatively low (see Supplementary Methods) it is possible that resource limitation was not a major constraint for individuals of any SES, and this may explain the lack of an effect of SES in previous studies. Another possible caveat of our study is its observational nature. Strong causal inference requires experimental manipulation, which is rarely possible in studies on natural populations. Nevertheless, several lines of evidence support our suggestion that sex-specific costs of reproduction is a driver of sexual dimorphism in lifespan. First, the gradual change in sexual dimorphism in lifespan in the population is accompanied by a concomitant decrease in average fertility. Second, we demonstrate a cost of reproduction in terms of reduced post-reproductive lifespan in females but not in males. Third, females showed a more pronounced increase in lifespan over time relative to males. Taken together, these observations strongly suggest that costs of reproduction can influence sexual dimorphism in lifespan.

In conclusion, we found a shift from a significantly female-biased to a significantly male-biased mortality during a period of only one century in Utah. We show that this shift may partly be explained by the decreased costs of reproduction in females during the demographic transition. This illustrates the importance of considering biological factors when elucidating the causes of shifting mortality patterns in human populations. Our results have implications for demographic forecasts, because fertility patterns and expected lifespans are continuously changing throughout the world. Further, as populations show shifting age-distributions towards older age classes and average lifespans continue to increase, biologically rooted differences in lifespan in the two sexes can have important implications for the development of strategies to achieve heathy ageing.

## Methods

### Data

The Utah Population Database (UPDB) is one of the most comprehensive computerised genealogies in the world. We included individuals that were born between 1820 and 1920 and had full known reproductive histories, as well as full information on several individual level control variables: married polygynously (yes/no), born in Utah (yes/no), birth order (firstborn son, firstborn daughter, laterborn of either sex, to control for effects of inherited wealth), and the identity of the birth mother (to account for non-independence of individuals born in the same family). This resulted in a total sample size of 75,667 reproducing females and 64,933 reproducing males.

### Sexual dimorphism in lifespan

We plotted Kaplan-Meir survival curves for the two sexes in four birth cohorts of 25 years each, including only individuals surviving to reproductive age. We tested for significant sex differences with log-rank and Wilcoxon tests in each cohort (Fig. 1). We then proceeded with parametric accelerated failure time (AFT) survival models, that included as fixed effects polygamy status, birth in or outside of Utah, birth order and birth cohort. Sex was added as a stratified fixed effect to allow different baseline survival shapes for the two sexes. Observations were clustered by maternal identity. The interaction between sex and birth cohort indicates whether the two sexes differ in each cohort. Mortality during migration to Utah is likely to have had sex-specific effects on survival, we therefore tested for a healthy migrant effect with parametric survival models.

### Reproduction-lifespan trade-off

We investigated the relationship between number of children born and post-reproductive lifespan with two approaches. First, in a Bayesian mixed-effects modelling framework. To focus on post-reproductive lifespan, individuals were required to live until age 55 or older. Models controlled for fixed effects as described above and maternal identity was included as a random effect. The initial model included the interaction between sex and both the linear and the quadratic (non-linear) term for number of children born. This was not significant and subsequently excluded from the model. Alternatively, post-reproductive lifespan can be analysed in a survival model framework, we thus applied parametric survival models to the same subset as above. We grouped the number of children born into six different levels representing low to high reproductive investment to obtain acceleration factors for different levels of reproductive output. Additional methods are available in Supplemental Methods.

## Additional Information

**How to cite this article**: Bolund, E. *et al*. Reduced costs of reproduction in females mediate a shift from a male-biased to a female-biased lifespan in humans. *Sci. Rep*. **6**, 24672; doi: 10.1038/srep24672 (2016).

## Supplementary Material

Supplementary Information

## Figures and Tables

**Table 1:** Sex differences in lifespan over four birth cohorts in Utah.

Birth cohort	estimate	SE	z	p	AF
1820–1844	−0.12	0.004	−3.21	0.0013	0.99
1845–1869	0.0039	0.002	2.11	0.035	1.00
1870–1894	0.029	0.001	2.15	<0.0001	1.03
1895–1919	0.048	0.002	2.52	<0.0001	1.05

Shown are parameter estimates (estimate) with associated standard errors (SE), z and p-values for the interaction between each birth cohort and sex obtained from parametric survival models. The acceleration factor (AF) indicates the estimated mean survival time for females in relation to males in each cohort.

**Table 2:** Sex differences in the effect of levels of reproductive investment on lifespan in individuals that survived until age 55 in Utah.

Number of children	estimate	SE	z	p	AF
Females					
2–4	0.0053	0.002	2.68	0.007	1.005
5–8	−0.0090	0.002	−4.56	<0.0001	0.99
9–14	–0.028	0.002	−3.23	<0.0001	0.97
15–21	−0.042	0.006	−6.58	<0.0001	0.96
Males					
2–4	0.011	0.003	3.73	0.0002	1.011
5–8	0.0091	0.003	3.27	0.001	1.0092
9–14	0.0019	0.003	0.65	0.52	1.0018
15–21	0.0032	0.005	0.70	0.48	1.0032
22–64	−0.0074	0.008	−0.89	0.37	0.99

Shown are parameter estimates (estimate) with associated standard errors (SE), z and p-values for the effect of different levels of reproductive investment obtained from parametric survival models. The acceleration factor (AF) indicates the estimated mean survival time for individuals with increasing parity compared to individuals with one child.
