# Supplementary material for: Reduced costs of reproduction in females mediate a shift from a male-biased to a female-biased lifespan in humans
Source: Sci Rep. 2016 Apr 18;6:24672. doi: 10.1038/srep24672 (PMC4834564; doi:10.1038/srep24672)

Supplementary Methods, Tables and Figures for:

Reduced costs of reproduction in females mediate a shift from a male-biased to a female-biased lifespan in humans

Elisabeth Bolund, Virpi Lummaa, Ken R. Smith, Heidi Hanson, Alexei A. Maklakov

Supplementary Methods

Data

The Utah Population Database (UPDB) is one of the most comprehensive computerised genealogies in the world. Genealogy records are gathered and provided by the Genealogical Society of Utah and span the pioneer settlement era in Utah in the mid-1800s as well as their Utah descendants until the present day[1]. These records include information on fertility and mortality for parents and their children. Before 1890 polygamy was practiced by a minority of the population[2]. Non-paternity rates in Utah are lower than the US average[3]. The Utah population during the period studied here suffered less mortality than most comparable populations, especially at early ages[4].

For this study, we focussed on the time period surrounding the demographic transition and included individuals from the UPDB that were born between 1820 and 1920 (no individual born before 1920 was still alive at the end of data coverage for this study in 2012). We required full information for a number of variables that were entered as fixed and random effects in the models, namely reproductive history, whether the individual was involved in a

polygamous marriage (4.7% of females and 1.9% of males), whether the individual was born in Utah (54% of females and 52% of males), birth order in three levels denoting whether an individual was the firstborn son, the firstborn daughter, or laterborn of either sex (to control for effects of inherited wealth), and the identity of the birth mother (maternal identity, to account for non-independence of individuals born in the same family). All individuals with full information for these fixed and random effects also had known birth and death dates, hence censoring was not employed in survival models. Individuals were divided into four birth cohorts of 25 years each, commencing 1820. This resulted in a total sample size of 75,667 reproducing females and 64,933 reproducing males.

Because the dataset is built from descendant genealogies, it likely underrepresents individuals that are known to not reproduce. In the present study, 1.7% of married individuals were known to be nulliparous, likely representing a non-random subset of the population because involuntary childlessness may be more common in frail individuals. Because of the difficulty to achieve a representative subset of nulliparous individuals and our interest in the cost of increased parity and the effect of changes in parity on lifespan, we therefore focussed on only reproducing individuals in the main analyses. For comparison, we include Kaplan-Meier survivor curves for presumably nulliparous individuals of the two sexes over the studied time period in Supplementary Fig. 1. These should be interpreted with knowledge of the lowered accuracy of the data in these individuals, because individuals that were registered as non-reproducers may be so because of a lack of information regarding their reproduction.

Sexual dimorphism in lifespan

To look at the differences in survival patterns between the two sexes and how this changed over time, we used the *survival* package in R[5] to plot Kaplan-Meir survival curves for the two

sexes in four cohorts of 25 years each, starting at the year 1820. Because the interest was in adult survival and we focussed on only reproducing individuals, individuals that died before reproductive age are not included in the survival analyses. Hence, the first deaths occur at age 15. We tested if the sexes differed significantly with log rank and Wilcoxon tests in each cohort (Figure 1). We then proceeded with parametric accelerated failure time (AFT) survival models using the *survreg* function in the survival package. AFT models assume that the effects of covariates are multiplicative with respect to survival time. Parametric models require that a distribution is specified for the survival times. We compared the fit of exponential, Weibull, log-normal and log-logistic models using two approaches: graphically, by comparing the predicted estimates from the fitted model with the observed Kaplan-Meier estimate of the survivor function, and analytically, by comparing model fits with different distributions specified, using χ^2-tests for nested models (exponential, Weibull and log-logistic) and using Akaikes information criterion (AIC) for non-nested models. This showed that a Weibull distribution provided the best fit. The Gompertz model was not intended to study mortality patterns beyond the age of about 80 years and thus was not considered[6]. Models included as fixed effects polygamy status, birth in or outside of Utah, birth order and birth cohort. Sex was added as a stratified fixed effect to allow different baseline survival shapes for the two sexes. Observations were clustered by maternal identity to account for clustering within mothers. The interaction between sex and birth cohort indicates whether the two sexes differ in each cohort. To ensure that these results are not dependant on the arbitrary division of the data into 25 year birth cohorts, we repeated the analyses, first with the data divided into 5 cohorts of 20 years each, and second, with birth year treated as a continuous variable. Results were robust to these alternative model specifications (not shown). Mortality during migration to Utah is likely to have had sex-specific effects on survival. The analyses detailed above includes all individuals with known birth and death information, regardless of

whether they were born or died in Utah or elsewhere. Because all individuals in the first cohort were born outside Utah, most of these individuals suffered the hardships of migration, affecting the sexes differently and resulting in an overall more robust cohort surviving to old ages. Subsequent cohorts are composed of four categories: individuals that 1) were born elsewhere and migrated to Utah, 2) were born and died in Utah, 3) were born in Utah and died elsewhere and, 4) were born and died outside of Utah. Cohort 2 thus offers the opportunity to compare migrants into Utah with individuals that spent their entire lives in Utah during a time period when migration still imposed hardships (Supplementary Fig. 1a). To test for a healthy migrant effect, we used a parametric survival model that adjusted for polygamy status and birth order as fixed effects, and clustered the observations by maternal identity. Because the conditions during migration changed over time, we validated the use of cohort 2 for this comparison by comparing migrants in cohort 2 with migrants in cohort 1 (Supplementary Fig. 1b) and looked at the difference in survival between migrants in these two cohorts in a parametric survival model within each sex, with covariates as described above.

Reproduction-lifespan trade-off

To test for a trade-off between reproduction and lifespan in the two sexes, we investigated the relationship between number of children born and post-reproductive lifespan with two approaches. First, we employed the R-package *MCMCglmm*, which uses an iterative Bayesian approach[7] and provides meaningful error estimates for derived variables by direct sampling from the posterior distribution. Individuals were required to live until age 55 or older, in order to focus on the effect of reproduction on late life mortality (100% of all women and 96.8% of all men had finished reproduction by this age). This avoids confounding deaths caused by childbearing (Figure 1) with post-reproductive mortality.

Hence, we subtracted 55 years from the total lifespan to obtain the response variable post-reproductive lifespan. Our selection criteria restricted the total sample size to 118,911 individuals. Models controlled for a number of fixed effects as described above (polygamy status, whether the individual was born in Utah, birth order and birth cohort). Maternal identity was included as a random effect to account for non-independence of observations within families. Number of children born was standardised within sexes prior to the analysis (mean of zero; SD of one) and we used post-reproductive lifespan as a Gaussian response variable. The initial model included the interaction between sex and both the linear and the quadratic (non-linear) term for number of children born. The 95% Bayesian credibility intervals for the interaction term indicate whether the linear or quadratic slope of the relationship between number of children and post-reproductive lifespan differed significantly between the two sexes. The quadratic term was not significant and subsequently excluded from the model. Because the maximum number of children was 64 in males and 21 in females, we investigated whether males with extreme values influenced the result by removing the 282 males with 22 or more children, generally polygamist men. After this equalisation of potential reproductive effort in the two sexes, we re-estimated the above model. This did not influence the results so we present results from the full dataset. To obtain sex-specific estimates for the relationship between reproductive effort and lifespan, we re-estimated the model for each sex separately. In the sex-specific models, the quadratic effect was significant in both sexes and was thus retained in both models. Because males that continued to reproduce after age 55 might inflate the estimates, the male-specific model was re-estimated excluding the 3.2% of males that continued to reproduce after age 55. This did not influence the results so we present results from the full dataset. All models were run with a prior with V = 1 and a degree of belief parameter (nu) of 0.002. Convergence of runs was assessed by visual inspection of output plots.

Alternatively, post-reproductive lifespan can be analysed in a survival model framework. To increase comparability with the models on sexual dimorphism in lifespan, and to obtain sex-specific estimates of acceleration factors for individuals with different levels of reproductive output, we used parametric survival models using the function *survreg* in the R-package survival. The subset used for analyses was restricted to individuals that survived until age 55 and their lifespan was used as the response variable. A Weibull distribution provided the best fit to the data in both sexes. Models included fixed effects as above, observations were clustered by maternal identity and birth cohort was added as a stratified fixed effect to allow different baseline survival shapes for each birth cohort. To obtain acceleration factors for different levels of reproductive output, we grouped the number of children born into six different levels representing low to high reproductive investment: one child, two to four children, five to eight children, nine to 14 children, 15 to 21 children and 22 to 64 children (males only).

Infant mortality may influence reproductive decisions and thus affect costs of reproduction, potentially in a sex-specific manner. The main cause of infant mortality in historical Utah was infectious diseases[4] and it is thus possible that infant mortality could affect the health and survival of mothers and fathers differently. While infant mortality in historical Utah was comparatively low, in the current study, 33% of reproducing women with full known reproductive history from birth cohorts 1 and 2 (that reproduced when fertility rates were high, Figure 1) lost one or more infants during the first year of life, while this dropped to 23% for women from cohorts 3 and 4 (that reproduced when fertility rates were lower). To account for the effects of infant mortality, we repeated the analyses of costs of reproduction with infant loss included as a two-level covariate (the individual had one or more children that

died during the first year of life versus all children survived their first year of life). This did not influence the results (not shown) and we thus present models excluding this variable, because the predictions regarding how infant mortality is expected to influence total fertility and sex-specific survival patterns are not straightforward.

References

1 Albright, L. A. C. Utah family-based analysis: Past, present and future. Hum Hered 65, 209-220, doi:10.1159/000112368 (2008).

2 Bean, L. L. & Mineau, G. P. The polygyny-fertility hypothesis - a re-evaluation. Pop Stud-J Demog 40, 67-81, doi:10.1080/0032472031000141846 (1986).

3 Bean LL, Mineau GP & DL, A. Fertility change on the American frontier: Adaptation and innovation. (University of Californa Press, 1990).

4 Bean, L. L. et al. Infant deaths in Utah, 1850-1939. Utah historical quarterly. 70, 16 (2002).

5 A package for survival analysis in S v. R package version 2.37-7 (2014).

6 Olshansky, S. J. & Carnes, B. A. Ever since Gompertz. Demography 34, 1-15, doi:10.2307/2061656 (1997).

7 Hadfield, J. MCMC methods for multi-response generalized linear mixed models: The MCMCglmm R package. J Stat Software 33, 1-22, doi:10.18637/jss.v033.i02 (2010).

Supplementary tables and figures

Table S1. Averages (± SD) and sample sizes of number of children and lifespan in the two sexes in four birth cohorts in historical Utah.

Birth cohort	Average children ± SD		Average lifespan ± SD		N	
	females	males	females	males	females	males
1820 to 1844	8.5 ± 3.3	11.1 ± 5.7	68.3 ± 17.8	70.1 ± 14.3	3298	3793
1845 to 1869	7.8 ± 3.3	8.3 ± 3.8	69.5 ± 17.5	70.2 ± 14.6	17331	16403
1870 to 1894	5.8 ± 3.1	5.8 ± 3.0	72.4 ± 18.1	70.8 ± 15.6	37156	33919
1895 to 1919	4.2 ± 2.5	4.2 ± 2.4	76.7 ± 17.5	72.6 ± 15.7	17882	10818

Table S2. Proportion of reproducing women who died the same year in which they gave birth to their last child, in four birth cohorts in historical Utah. Sample sizes as reported in Table S1.

Birth cohort	1820 to 1844	1845 to 1869	1870 to 1894	1895 to 1919
Proportion deceased	0.0633	0.053	0.037	0.019

Figure S1.

Comparison of sex-specific survival patterns between (a) individuals that migrated into Utah and individuals that spent their entire lives in Utah in birth cohort (coh) 2 and (b) individuals that migrated into Utah in birth cohort 1 compared to migrants in birth cohort 2. Birth cohort 2 is composed of four groups of individuals with respect to migratory status: individuals that were born and died in Utah, individuals that were born elsewhere and died in Utah, individuals that were born in Utah but died elsewhere and individuals that spent their entire lives outside Utah. In (a), the first two of these groups are compared, separately in the two sexes. The early migrants to Utah faced harsher conditions during migration than later migrants. Therefore, figure (b) compares the effects of migration on survival in the early migrants into Utah of cohort 1 with the later migrants of cohort 2, separately in the two sexes. Note that migrants into Utah in cohort 2 thus appear in both figures, males in light blue and females in orange. In (a), these migrants are compared to non-migrants within the same cohort (males in blue and females in red), whereas in (b), the migrants in cohort 2 are compared to migrants in cohort 1 (males in blue and females in red). Shown are the birth years (Yr) contained in birth cohort 1 (a) or 1 and 2 (b) and the sample size in each group (N). Dotted vertical lines indicate the average lifespan of (a) non-migrants (black) and migrants (grey) in both sexes pooled and of (b) migrants in cohort 1 (black) and cohort 2 (grey). All p-values were > 0.2 from GW and LR tests of the difference between individuals within each sex that were born in Utah and individuals that migrated into Utah (a), and likewise (b), all p-values comparing migrants in cohort 1 to migrants in cohort 2 were > 0.25 in females. Migrant males in cohort 1 tended to live longer than migrant males in cohort 2, GW $\chi^2 = 9.3$, $p = 0.002$, LR $\chi^2 = 3.7$, $p = 0.053$.

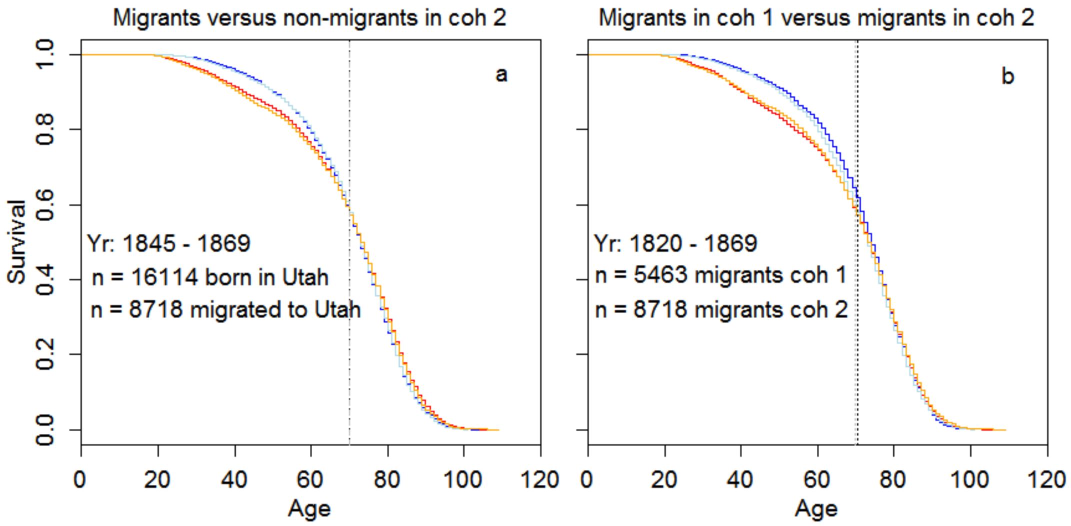
Migrants versus non-migrants in coh 2
Migrants in coh 1 versus migrants in coh 2
a
b
Survival
1.0
0.8
0.6
0.4
0.2
0.0
Yr: 1845 - 1869
n = 16114 born in Utah
n = 8718 migrated to Utah
Yr: 1820 - 1869
n = 5463 migrants coh 1
n = 8718 migrants coh 2
0
20
40
60
80
100
120
Age

208

Figure S2.

Age-specific adult survival for females (red) and males (blue) that presumably did not reproduce, divided into four 25-year birth cohorts that cover the demographic transition in Utah. Shown in each figure are the birth years contained in that cohort (Yr), the sample size (n), the average ± SD number of children born to females (CHf), the χ^2 and p-values indicating differences in the survivor function between the two sexes from the Peto and Peto modification of the Gehan-Wilcoxon test (GW, this test weights differences in survivorship that occur early more heavily than differences at later survival times) and from a log-rank test (LR, this test weights differences at later survival times more heavily). Dotted vertical lines indicate the average lifespan of each sex in each cohort.

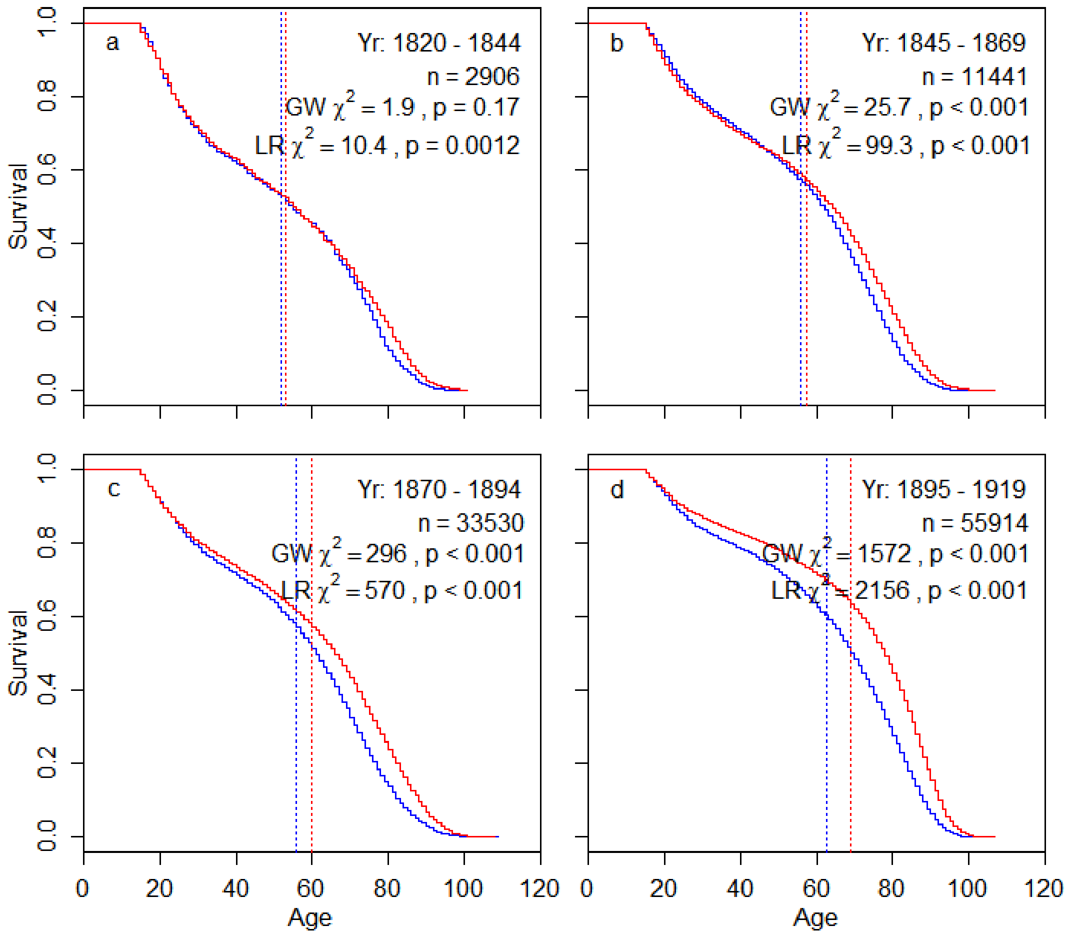

Supplement: Supplementary Information [file srep24672-s1.pdf]
